# General instability of dipeptides in concentrated sulfuric acid as relevant for the Venus cloud habitability

**DOI:** 10.1038/s41598-024-67342-w

**Published:** 2024-07-24

**Authors:** Janusz J. Petkowski, Maxwell D. Seager, William Bains, Sara Seager

**Affiliations:** 1 Department of Earth, Atmospheric and Planetary Sciences, Massachusetts Institute of Technology, 77 Massachusetts Avenue, Cambridge, MA 02139 USA; 2 Faculty of Environmental Engineering, Wroclaw University of Science and Technology, 50-370 Wroclaw, Poland; 3 JJ Scientific, 02-792 Mazowieckie, Warsaw, Poland; 4 Department of Chemistry and Biochemistry, Worcester Polytechnic Institute, Worcester, MA 01609 USA; 5 Nanoplanet Consulting, Concord, MA 01742 USA; 6 School of Physics and Astronomy, Cardiff University, 4 The Parade, Cardiff, CF24 3AA UK; 7 Rufus Scientific, Melbourn, Herts SG8 6ED UK; 8 Department of Physics, Massachusetts Institute of Technology, 77 Massachusetts Avenue, Cambridge, MA 02139 USA; 9 Department of Aeronautics and Astronautics, Massachusetts Institute of Technology, 77 Massachusetts Avenue, Cambridge, MA 02139 USA

**Keywords:** Astrobiology, Astrobiology

## Abstract

Recent renewed interest in the possibility of life in the acidic clouds of Venus has led to new studies on organic chemistry in concentrated sulfuric acid. We have previously found that the majority of amino acids are stable in the range of Venus’ cloud sulfuric acid concentrations (81% and 98% w/w, the rest being water). The natural next question is whether dipeptides, as precursors to larger peptides and proteins, could be stable in this environment. We investigated the reactivity of the peptide bond using 20 homodipeptides and find that the majority of them undergo solvolysis within a few weeks, at both sulfuric acid concentrations. Notably, a few exceptions exist. HH and GG dipeptides are stable in 98% w/w sulfuric acid for at least 4 months, while II, LL, VV, PP, RR and KK resist hydrolysis in 81% w/w sulfuric acid for at least 5 weeks. Moreover, the breakdown process of the dipeptides studied in 98% w/w concentrated sulfuric acid is different from the standard acid-catalyzed hydrolysis that releases monomeric amino acids. Despite a few exceptions at a single concentration, no homodipeptides have demonstrated stability across both acid concentrations studied. This indicates that any hypothetical life on Venus would likely require a functional substitute for the peptide bond that can maintain stability throughout the range of sulfuric acid concentrations present.

## Introduction

We are motivated to study organic chemistry in concentrated sulfuric acid to support the notion that organic molecules can survive in such a harsh environment. This in turn is inspired by the speculation of the potential habitability of Venus (e.g.^1–11^), not at the 700 K surface, but in liquid droplets of the cloud layers located at 48–60 km altitudes, where temperatures match those found on Earth’s surface^12^. While complex organic chemistry does not equate to life, it forms a foundation for habitability^13^. An environment that supports the origin and persistence of complex organic chemistry is potentially habitable. Therefore, investigating the stability of complex organic molecules in concentrated sulfuric acid environments is a critical step towards identifying whether clouds of Venus, so fundamentally different from Earth’s conditions, could be habitable.

Recent research challenges the prevailing view in planetary science that organic chemistry cannot exist in the aggressive solvent sulfuric acid^14,15^. Liquid concentrated sulfuric acid, the predominant component of the Venus clouds (and perhaps on certain exoplanets^16^), may host a diverse array of organic reactions potentially supportive of life forms unlike those on Earth. Such organic reactions have even been used in industry. For example the oil refinement industry uses concentrated acid to process crude oil and as a result generates “red oil”, an unwanted byproduct rich in various organic compounds, including aromatics dissolved in the acid^17–19^. Such reactivity indicates a broader potential for organic chemistry in such environments. Moreover, pioneering work by Spacek and Benner demonstrated that a rich organic chemistry can spontaneously arise in concentrated sulfuric acid from simple precursors such as formaldehyde and CO^20–23^. Our group has extended these findings, showing the stability of nucleic acid bases and their core structures in concentrated sulfuric acid at room temperature^24,25^.

The stability of simple organic molecules in recent studies is promising, but life requires more structurally complex molecules for biological function. For example, life fundamentally relies on enzymes for survival. Enzymes are specialized catalysts that accelerate biochemical pathways by factors exceeding 10^10^ fold (e.g.^26–28^). Without enzymes, simple processes, such as metabolizing a single glucose molecule would take an extraordinary amount of time. On modern Earth, enzymes are proteins, built from a set of 20 biogenic amino acids, which are joined together by peptide bonds to form linear polypeptides. The polypeptides fold into 3D protein structures determined by their amino acid sequence. The peptide bond is central to the integrity of a protein. Study of the reactivity of the peptide bond is therefore a necessary first step to understand how a protein might behave in concentrated sulfuric acid.

We have previously shown that the “amino acid backbone” is stable in concentrated sulfuric acid (at 98% w/w and 81% w/w, the rest being water) at room temperature^29^. Furthermore, we found that the majority of the amino acid side chains were unreactive, with the remainder largely found to be stable after chemical modification of the side chain. The natural next question relates to the stability of dipeptides, as precursors to larger peptides and proteins in concentrated sulfuric acid. Early studies suggest that some peptide bonds and peptide chains are in fact remarkably stable in this aggressive solvent (e.g.^30–36^).

We therefore aim to address the stability of the peptide bond on a well-defined, finite set of dipeptides. The peptide bond is generally known to be readily hydrolyzed in acidic aqueous conditions (e.g.^37–39^). However, concentrated sulfuric acid is not an aqueous solvent, so it is important to know to what extent this solvolysis is universal, and if there are any dependencies of peptide bond reactivity based on the residues that form it. Long-term stability of the peptide bond is crucial if protein-like polymers are to be used in a sulfuric acid biochemistry.

We use nuclear magnetic resonance (NMR) to assess the stability of the peptide bond in liquid sulfuric acid concentrations in the range relevant for the clouds of Venus at room temperature over timescales of days to four months. We present our results in “Results: reactivity and stability of dipeptides in concentrated sulfuric acid” section followed by a discussion in “Discussion” section.

## Results: reactivity and stability of dipeptides in concentrated sulfuric acid

Our main finding is that the peptide bond is generally unstable in concentrated sulfuric acid.

In the lowest concentrations of sulfuric acid found in the Venus clouds (81% w/w), acid-catalyzed hydrolysis breaks the peptide bond. The unstable dipeptides are typically hydrolyzed after a few days. However, there are a few stable exceptions—the dipeptides containing amino acids with small aliphatic hydrophobic side-chains.

In the highest concentrations of sulfuric acid found in the Venus clouds (98% w/w) the dipeptides are largely unstable. Our major finding is that the solvolysis of the peptide bond in 98% w/w sulfuric acid is not the typical acid-catalyzed hydrolysis. We have also found two stable homodipeptide exceptions. One exception is GG, which is stable for over four months, likely due to lack of a side chain on the alpha carbon. HH is stable for months as well, possibly due to the bulky positively charged side chains that hinder the solvolytic action of concentrated sulfuric acid.

### Acid-catalyzed hydrolysis in 81% w/w concentrated sulfuric acid

**Figure 1:**
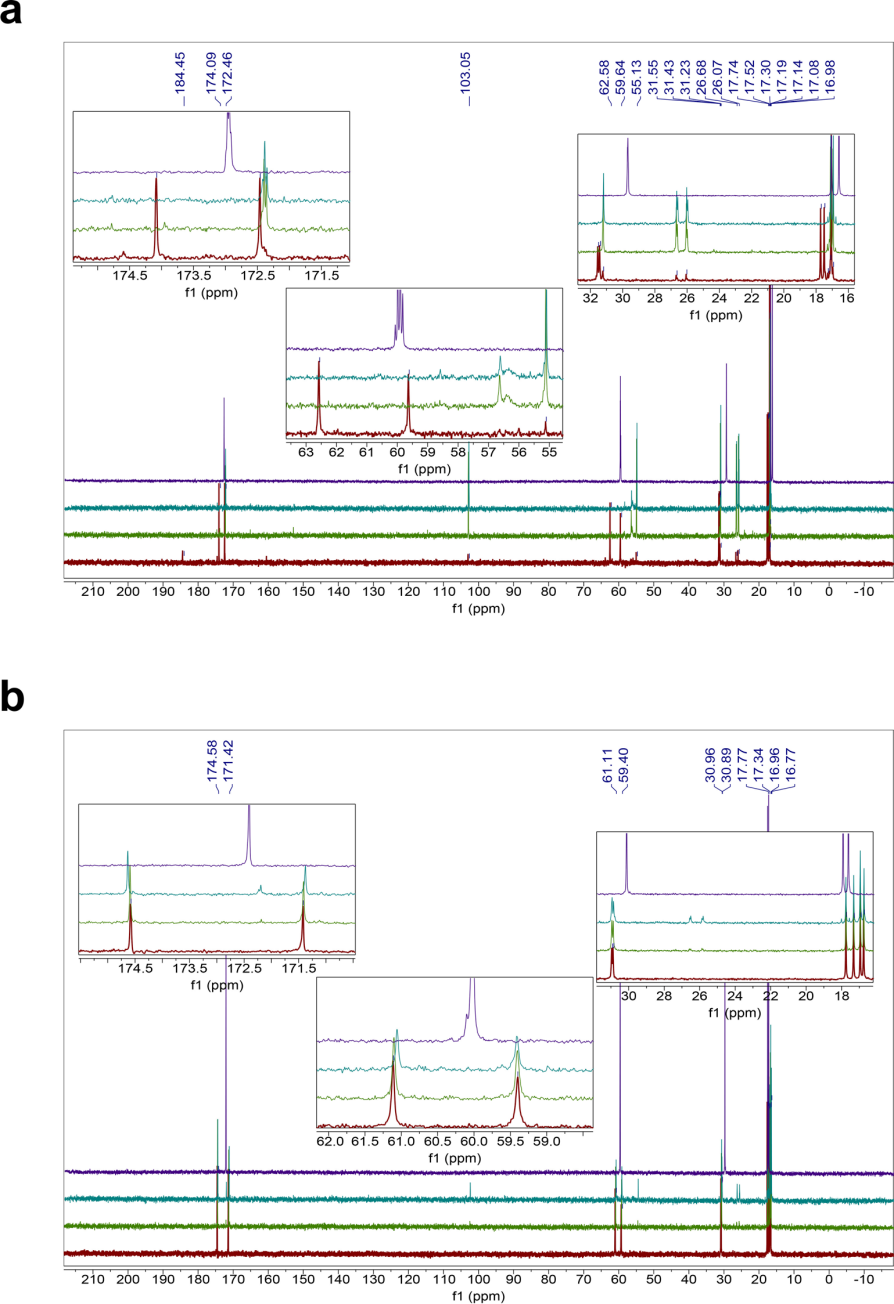
Reactivity of the VV homodipeptide in concentrated sulfuric acid. Time series of ^13^C NMR spectra of the VV with colors indicating the time incubated in concentrated sulfuric acid at room temperature: red = 1 day, green = 5 weeks, teal = 4 months. (**A**) The VV dipeptide incubated in 98% w/w sulfuric acid shows solvolysis without the release of the component single valine residue, as seen by comparison to the NMR spectra of the 1-month-incubated single amino acid valine (purple curve)^29^. (**B**) The VV dipeptide incubated in 81% w/w sulfuric acid. VV is stable for at least 5 weeks, as seen by comparison the NMR spectra of the 1-month-incubated single amino acid valine (purple curve)^29^ but eventually shows possible signs of reactivity after many months in 81% w/w sulfuric acid.

**Figure 2:**
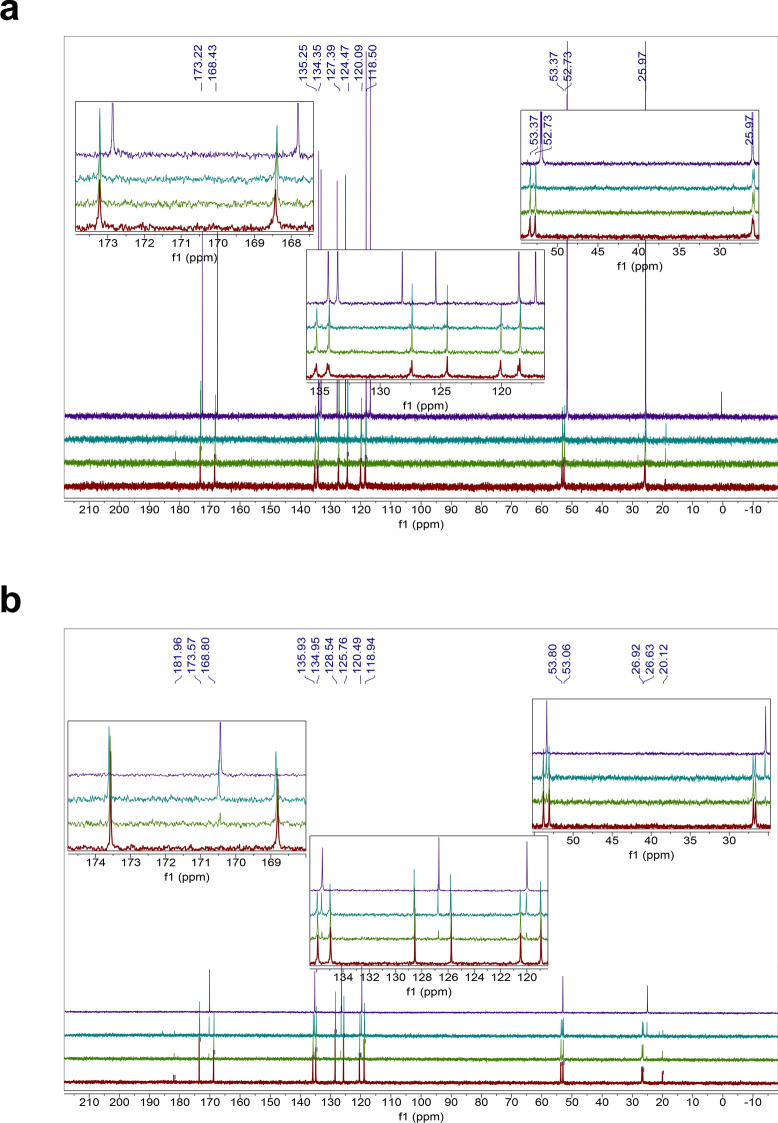
Stability of the HH homodipeptide in concentrated sulfuric acid. Time series of ^13^C NMR spectra of the HH with colors indicating the time incubated in concentrated sulfuric acid at room temperature: red = 1 day, green = 5 weeks, teal = 4 months. Note that additional small peaks around 1 ppm and 20 ppm are leftover impurities from peptide synthesis and not a sign of reactivity of the HH dipeptide. (**A**) The HH is stable in 98% w/w sulfuric acid for at least 4 months, as seen by comparison with the NMR spectra of the HH dipeptide in D_2_O (purple curve). (**B**) The HH dipeptide incubated in 81% w/w sulfuric acid. HH is unstable in 81% w/w sulfuric acid and undergoes slow hydrolysis after 5 weeks as seen by the comparison to the NMR spectra of the 1-month-incubated single amino acid histidine (purple curve)^29^.

**Figure 3:**
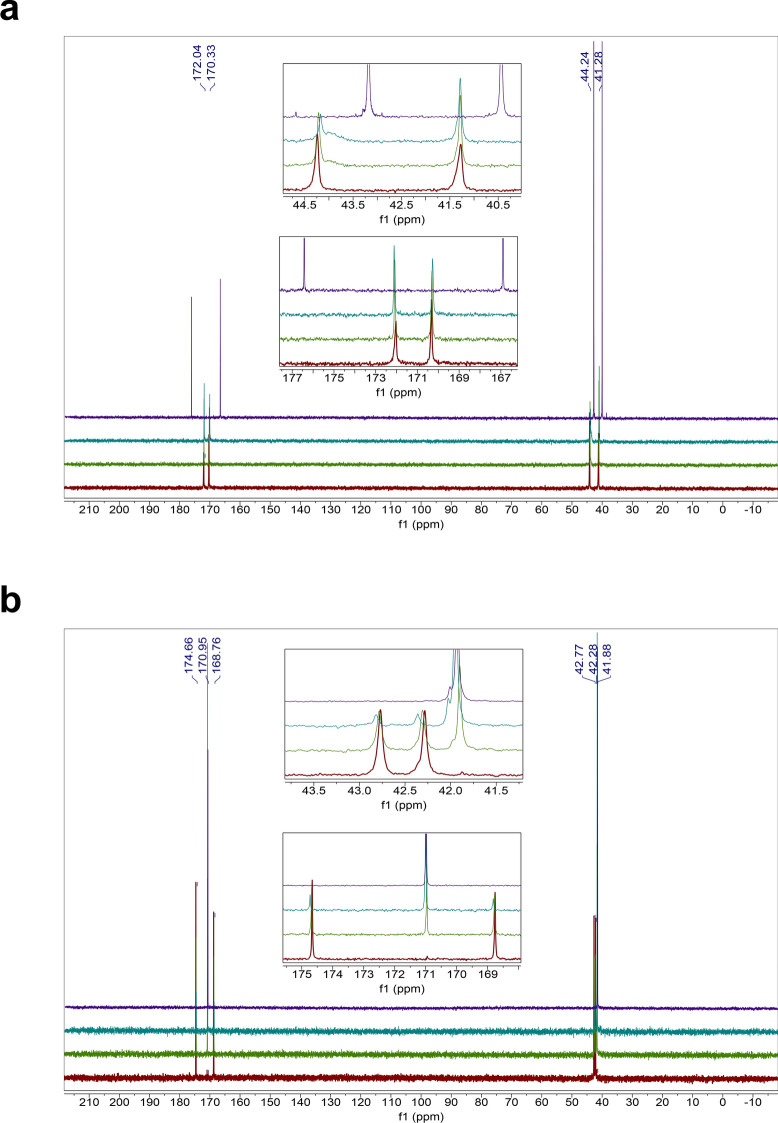
Stability of the GG homodipeptide in concentrated sulfuric acid. Time series of ^13^C NMR spectra of the GG with colors indicating the time incubated in concentrated sulfuric acid at room temperature: red = 1 day, green = 5 weeks, teal = 4 months. (**A**) The GG dipeptide is stable in 98% w/w sulfuric acid for at least 4 months, as seen by comparison with the NMR spectra of the GG dipeptide in D_2_O (purple curve). (**B**) The GG dipeptide incubated in 81% w/w sulfuric acid. GG is unstable in 81% w/w sulfuric acid and undergoes hydrolysis after 1 day, as seen by the comparison to the NMR spectra of the 1-month-incubated single amino acid glycine (purple curve)^29^.

**Figure 4:**
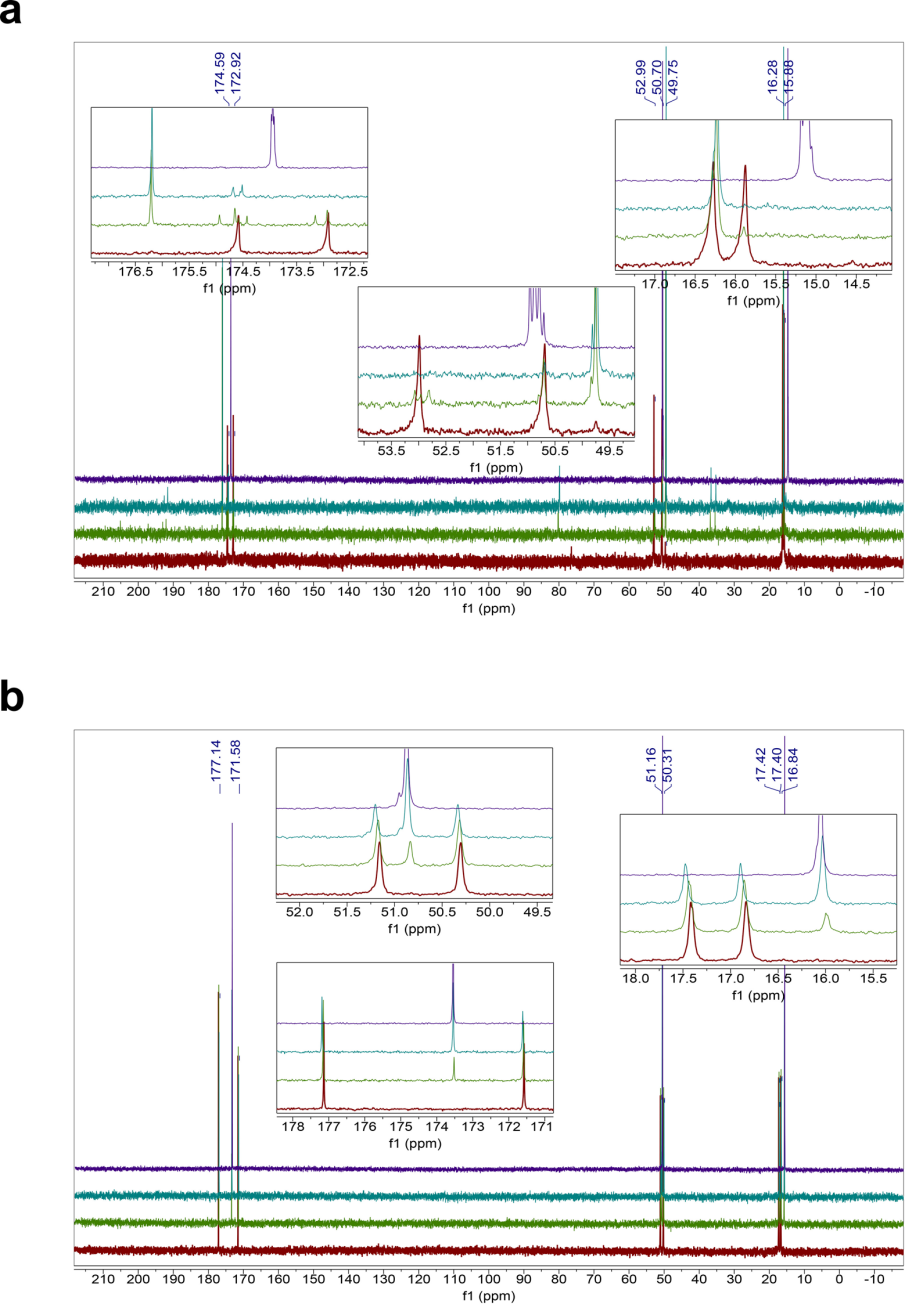
Reactivity of the AA homodipeptide in concentrated sulfuric acid. Time series of ^13^C NMR spectra of the AA with colors indicating the time incubated in concentrated sulfuric acid at room temperature: red = 1 day, green = 5 weeks, teal = 4 months. (**A**) The AA dipeptide incubated in 98% w/w sulfuric acid shows solvolysis without the release of the single monomeric alanine residue, as seen by comparison to the NMR spectra of the 1-month-incubated single amino acid alanine (purple curve)^29^. (**B**) The AA dipeptide incubated in 81% w/w sulfuric acid. AA is unstable in 81% w/w sulfuric acid and undergoes hydrolysis after 1 day as seen by the comparison to the NMR spectra of the 1-month-incubated single amino acid alanine (purple curve)^29^.

**Figure 5:**
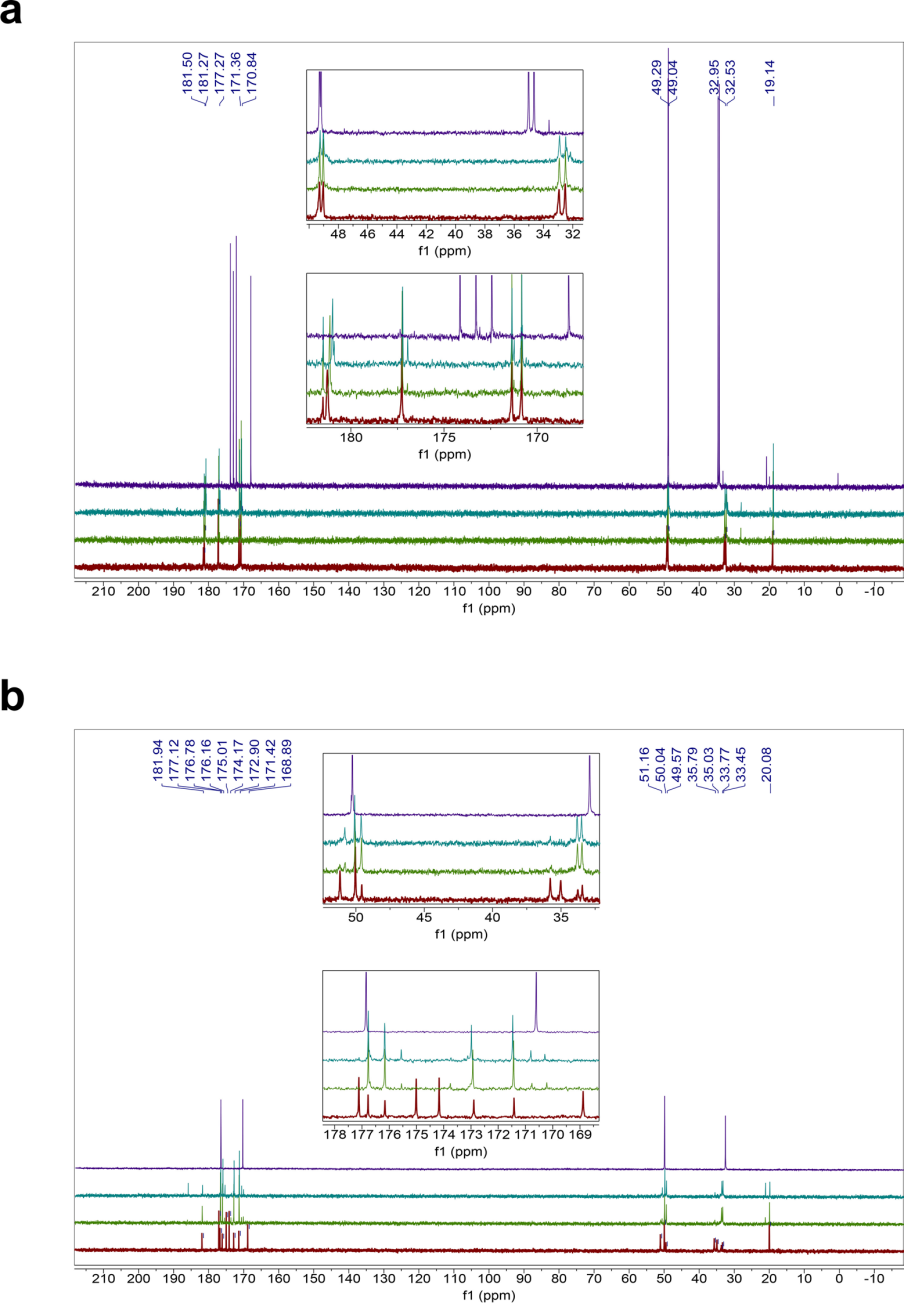
Reactivity of the DD homodipeptide in concentrated sulfuric acid. Time series of ^13^C NMR spectra of the DD with colors indicating the time incubated in concentrated sulfuric acid at room temperature: red = 1 day, green = 5 weeks, teal = 4 months. Note that additional small peaks around 1 ppm and 20 ppm are leftover impurities from peptide synthesis and not a sign of reactivity of the DD dipeptide. (**A**) The DD is stable in 98% w/w sulfuric acid for at least 5 weeks, as seen by comparison with the NMR spectra of the DD dipeptide in D_2_O (purple curve), but eventually slowly degrades after few months. (**B**) The DD dipeptide incubated in 81% w/w sulfuric acid. DD is unstable in 81% w/w sulfuric acid undergoes possible cyclization and hydrolysis, as seen by the comparison to the NMR spectra of the 1-month-incubated single amino acid aspartic acid (purple curve)^29^.

**Figure 6:**
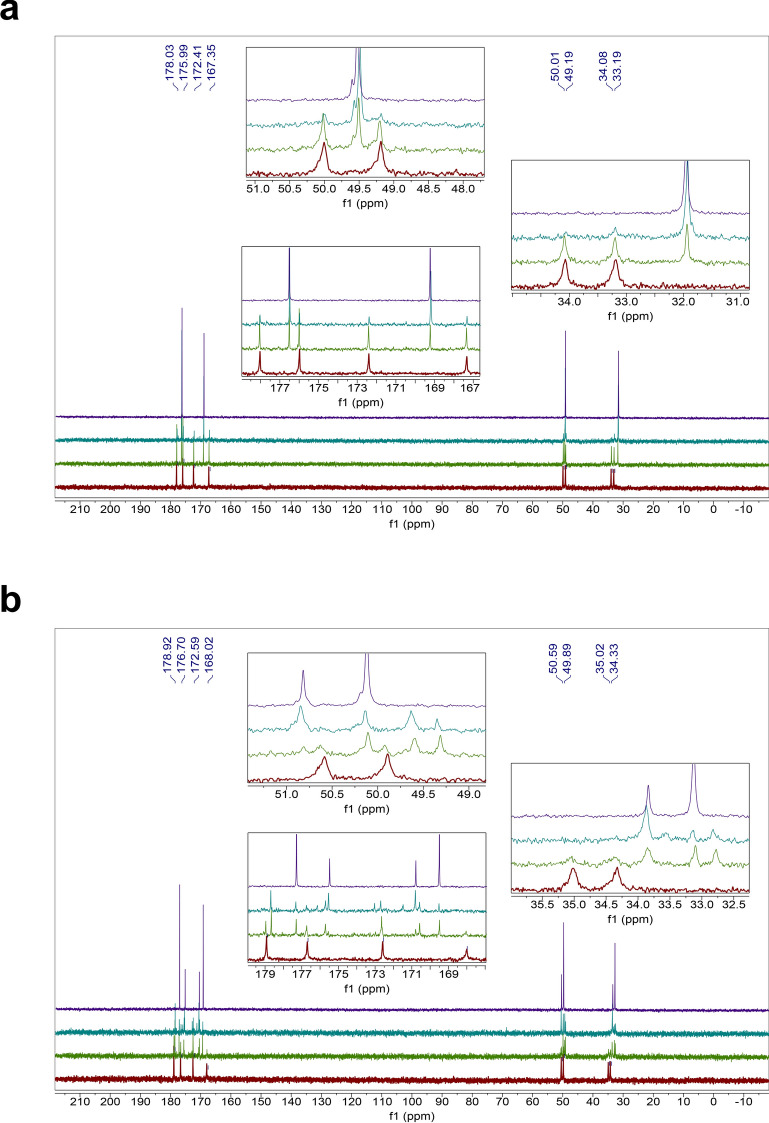
Reactivity of the NN homodipeptide in concentrated sulfuric acid. Time series of ^13^C NMR spectra of the NN with colors indicating the time incubated in concentrated sulfuric acid at room temperature: red = 1 day, green = 5 weeks, teal = 4 months. (**A**) The NN dipeptide is reactive in 98% w/w sulfuric acid and undergoes C-terminal Asn-meditated peptide bond cleavage and the release of free asparagine residue, as seen by comparison the NMR spectra of the 1-month-incubated single amino acid asparagine (purple curve)^29^. (**B**) The NN dipeptide incubated in 81% w/w sulfuric acid. NN is unstable in 81% w/w sulfuric acid and undergoes deamidation and hydrolysis after 1 day, as seen by the comparison to the NMR spectra of the 1-month-incubated single amino acid asparagine (purple curve)^29^.

The majority of the homodipeptides are unstable in 81% w/w concentrated sulfuric acid. This is expected due to the well-known acid-catalyzed hydrolysis of the peptide bond (e.g.^37,38^). Our evidence is that the breakdown products are the native monomeric amino acids of each dipeptide, seen via ^1^H and ^13^C NMR, identified using our library of single amino acid in in D_2_SO_4_ NMR spectra^29^. The timescale of instability is modulated by the amino acid side chains (Table 1). (Figs. 1, 2, 3, 4, 5, 6) (Figs. S1–S4).

**Table 1:**
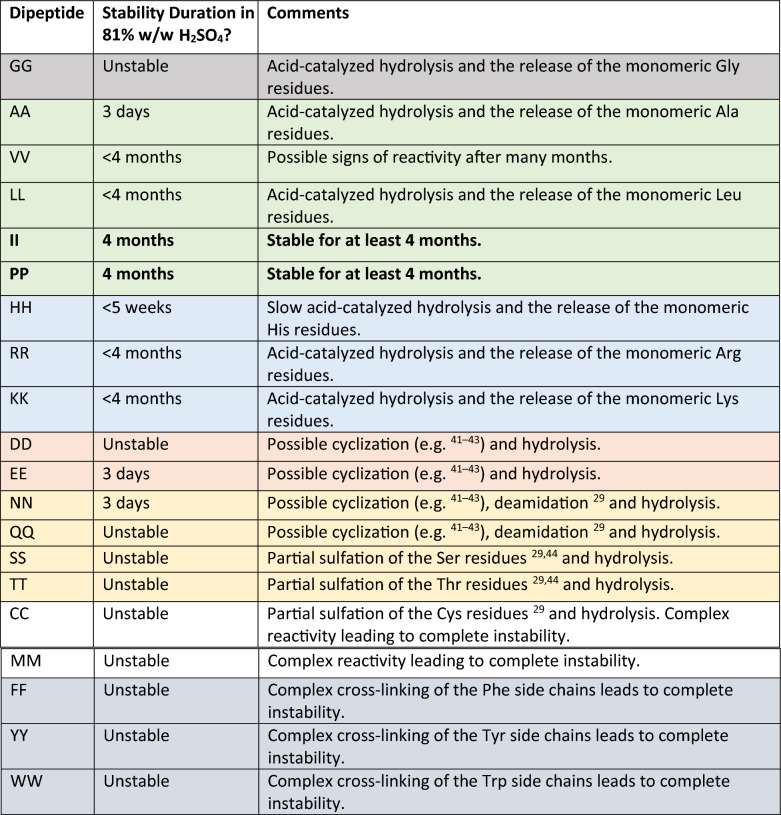
Stability and reactivity of homodipeptides in 81% w/w sulfuric acid.

The homodipeptides are color-coded by the type of amino acid side chains. Grey: glycine (G); Green: small hydrophobic amino acids (A, V, L, I, P); Blue: positively-charged amino acids (H, R, K); Pink: negatively-charged amino acids (D, E); Yellow: uncharged polar amino acids (N, Q, S, T); White: sulfur-containing amino acids (C, M); Silver: aromatic amino acids (F, Y, W). Unstable means near immediate instability. Bold face indicates homodipeptides that are stable.

The homodipeptides fall into reactivity categories (Table 1). Those with small hydrophobic side chains (VV, LL, II, PP) are stable to acid-catalyzed hydrolysis in 81% w/w sulfuric acid for up to 5 weeks of incubation at room temperature (Table 1, Fig. 1). The ^13^C NMR spectra collected after 1 day incubation in 81% w/w sulfuric acid match the spectra collected after 5 week incubation, with no new peaks emerging, indicating long-term stability of the VV, LL, II, PP dipeptides. After 5 weeks the VV and LL dipeptides slowly break down. In contrast, the PP and II dipeptides appear to be stable to acid catalyzed hydrolysis for more than four months (up to the time of our final measurements). Their stability in 81% w/w acid likely results from the significant entropic effect, provided by the hydrophobic side chains. The hydrophobicity of the side chains creates unfavorable conditions for an attack by the OH^−^ ion on the peptide carbonyl in aqueous acid^40^.

The homodipeptides with positively charged amino acid side chains are also generally stable to hydrolysis over the span of weeks. The RR and KK dipeptides are stable for 5 weeks in 81% w/w sulfuric acid, while HH undergoes a very slow acid-catalyzed hydrolysis with the release of the monomeric histidine residue after 5 weeks (Fig. 2). The RR and KK dipeptides eventually do hydrolyze to single amino acids as seen after a four-month long incubation in 81% w/w sulfuric acid.

The homodipeptides that are unstable in 81% w/w sulfuric acid undergo different types of reactivity (Table 1). For example, GG and AA dipeptides follow the classical acid-catalyzed hydrolysis of the peptide bond that depends on the presence of significant water content^37,39^. As expected, this reactivity results in the release of original single amino acids, although the efficiency of the hydrolysis differs with respect to the homodipeptide amino acid composition (Figs. 2, 3, 4). The dipeptides with negatively charged side chains (DD and EE) or dipeptides with uncharged polar residues (NN and QQ) undergo a possible cyclization reaction (e.g.^41–43^) and subsequent hydrolysis (Figs. 5, 6). The SS and TT homodipeptides get sulfated in concentrated sulfuric acid^29,44^ and eventually hydrolyze. Five dipeptides (CC, MM, FF, YY, WW) undergo reactivity in 81% w/w sulfuric acid that results in their instability and breakdown to a complex mixture of products after less than 1 day of incubation.

### A new solvolysis mechanism in 98% w/w concentrated sulfuric acid

The large majority of homodipeptides are unstable in 98% w/w sulfuric acid. Our major finding is that the solvolysis in 98% w/w results in different products than we find for the well-known acid-catalyzed hydrolysis in 81% w/w sulfuric acid. None of the breakdown products in 98% w/w sulfuric acid match the individual amino acids making up the dipeptides (with one exception, NN, discussed below). Here we use our previous work on the amino acids in concentrated sulfuric acid for interpretation of the solvolysis results^29^. We propose a solvolysis mechanism of the peptide bond in 98% w/w sulfuric acid in a separate paper^45^.

The solvolysis of AA and VV homodipeptides (Figs. 1, 4) are useful to illustrate the release of products different from the individual amino acids that make up the dipeptide because AA and VV have few carbon atoms making the NMR more easily interpretable than for other homodipeptides. The dominant products of the solvolysis of these homodipeptides appear to be chemically modified single amino acids with an additional complex mixture of minor products present in the solution. The number of carbon peaks of the dominant solvolysis product on the ^13^C NMR spectra matches the number of carbons of the single amino acid. However, the carbon peaks of the dominant product are shifted with respect to the corresponding peaks of the unmodified amino acid (Figs. 1, 4). This shift indicates that the dominant product of the solvolysis is a derivative of the single amino acid that builds the dipeptide.

We now turn to a description of reactivity categories of dipeptides in 98% w/w concentrated sulfuric acid (Table 2). We first highlight that GG is stable for months with no signs of reactivity. The reasons behind the remarkable stability of the GG peptide bond is a topic of a separate study^45^, but we speculate that the stability of GG dipeptide is due to lack of a side chain on the alpha carbon in glycine.

**Table 2:**
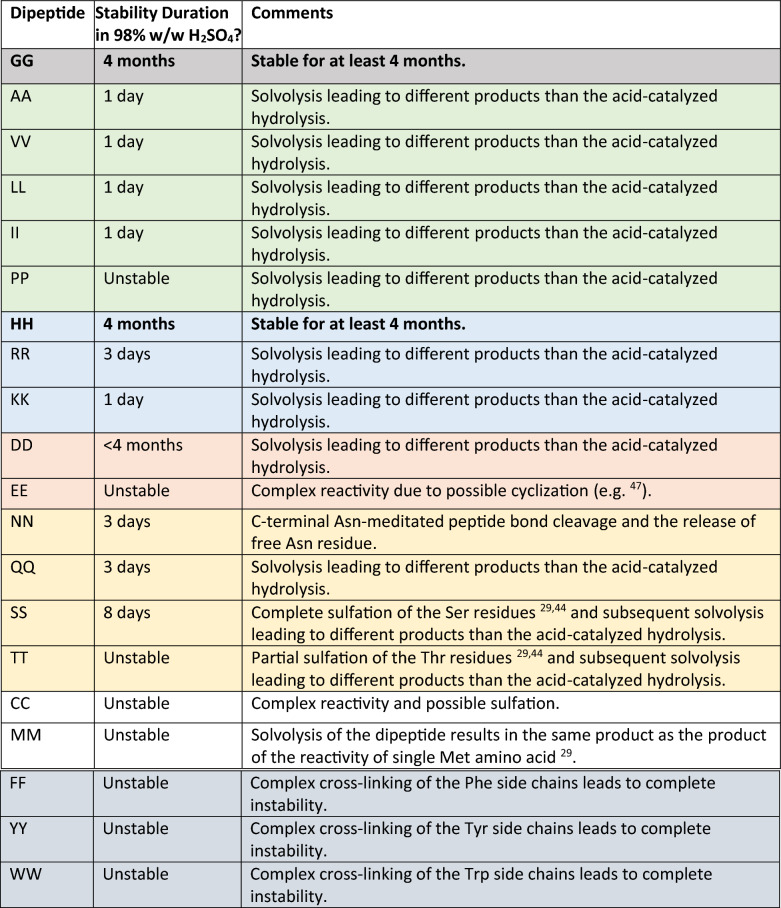
Stability and reactivity of homodipeptides in 98% w/w sulfuric acid.

The homodipeptides are color-coded by the type of amino acid side chains. Grey: glycine (G); Green: small hydrophobic amino acids (A, V, L, I, P); Blue: positively-charged amino acids (H, R, K); Pink: negatively-charged amino acids (D, E); Yellow: uncharged polar amino acids (N, Q, S, T); White: sulfur-containing amino acids (C, M); Silver: aromatic amino acids (F, Y, W). Unstable means near immediate instability. Bold face indicates homodipeptides that are stable.

The HH dipeptide also remains intact in 98% w/w sulfuric acid for up to our longest incubation time of 4 months (Fig. 2). We speculate that the bulky positively charged histidine side chains hinder the solvolytic action of concentrated sulfuric acid. Note that the well-known hydrolytic mechanism involving an attack by OH^−^ ion cannot happen in 98% w/w sulfuric acid due to negligible concentration of OH^−^ ions^46^. Other bulky aromatic residues (FF, YY and WW) cannot provide such a stabilizing effect because crosslinking reactivity of the sidechains results in rapid degradation of the dipeptide before peptide bond solvolysis, a reaction discussed further below.

The DD dipeptide also stands out, as it appears to be stable for weeks to months in 98% w/w sulfuric acid, with only a slight degradation visible after couple of months of incubation in sulfuric acid at room temperature (Table 2). As it is in the case of HH, it is unclear why the DD homodipeptide is so stable in 98% w/w sulfuric acid, but its stabilization to solvolysis could involve intramolecular interactions between the aspartic acid sidechains and the rest of the dipeptide that results in an inefficient solvolysis of the DD dipeptide.

Some of the homodipeptides undergo complex reactivity in 98% w/w sulfuric acid. For example, we hypothesize that the EE homodipeptide likely undergoes cyclization (as suggested by our NMR and previous studies (e.g.^47^)) and subsequent solvolysis, while the SS homodipeptide gets fully sulfated before complete solvolysis (Table 2).

The reactivity of the NN homodipeptide in 98% w/w sulfuric acid is an interesting exception to the solvolysis that for all other homodipeptides does not result in the release of the native amino acids. The NN dipeptide undergoes an autocatalyzed solvolysis of the peptide bond with the release of a single asparagine amino acid that is stable to further reactivity (Fig. 6). The Asn-dependent cleavage of the peptide bond is well known and happens in water in vitro and in vivo^48–51^.

Five homodipeptides (CC, MM, FF, YY, WW) degrade completely, in less than 1 day, upon incubation in 98% w/w sulfuric acid. We expect that the degradation of the aromatic homodipeptides (FF, YY, WW) is, at least in part, due to cross-linking and subsequent reactivity of the side-chains, as similar chemistry occurs during “red oil” formation^17–19^. Solutions containing FF, YY, and WW dipeptides turn brown upon addition to 98% w/w sulfuric acid, and eventually after 1–2 weeks, into a completely opaque black solution. Such color change is indicative of a well-known cross-linking reaction that happens in concentrated sulfuric acid^17–19^.

## Discussion

The amide bond, and the peptide bond in particular, is one of the most important and common bonds in biochemistry. The amide bond forms the backbone of proteins as well as scaffolds of countless small molecule metabolites. Since the formation of polymers is one of the requirements for any life, no matter its chemical makeup^52^, the chemical stability of the amide bond as a building block of polymers could greatly inform the potential habitability of liquid sulfuric acid as a solvent. In particular, understanding the reactivity of the peptide bond in concentrated sulfuric acid is crucial for the assessment of the possibility for the terrestrial biogenic amino acids to participate in the formation of complex polymeric structures of the hypothetical sulfuric acid biochemistry.

We show that the majority of peptide bonds in the tested homodipeptides undergo solvolysis within a few weeks time at both 98% w/w and 81% w/w sulfuric acid concentrations. We note that this is significantly less stable than in pure water. The peptide bond is very stable to non-catalyzed hydrolysis in pure aqueous solutions. The half-life of compounds containing peptide bonds may span several hundred years in water, at ambient temperature and pressure conditions^53^. Furthermore, there are no homodipeptides that are stable for more than five weeks at both acid concentrations. This lack of overlap in peptide bond stability in the range of sulfuric acid concentrations present in the liquid droplets of Venus’ clouds means it likely cannot be used by any hypothetical Venusian life forms that use concentrated sulfuric acid as a solvent. In other words, if there are no amino acid combinations that could form stable peptide bonds at the range of sulfuric acid concentrations present in the Venus’ clouds then the chances for peptides as main components of sulfuric acid biochemistry, especially if significant long-term stability is required, are low.

One might imagine the possibility of large-scale forces that hold proteins together overpowering potential peptide bond instability. Such stabilizing effects can be explored with modern computational methods and further experimental tests in concentrated sulfuric acid. We note however, that proteins are unlikely to be stable in concentrated sulfuric acid which is expected to disrupt proper folding of the polypeptide chain (at least for terrestrial proteins that have evolved to fold in water) (e.g.^54^).

If the peptide bond cannot be part of a hypothetical Venusian sulfuric acid biochemistry, then the immediate question is whether there are any other chemical functional groups that could act as a structural substitute for the peptide bond. Such peptide bond functional mimics would have to be capable of building complex polymeric structures that are stable over the range of temperatures and acid concentrations present in the entire Venusian cloud deck. Moreover, such a hypothetical substitute protein-like polymer should also have the capability to fold into diverse 3D structures. We stress here, that this does not necessarily mean creation of Earth-like protein polymers, or chains that are folded in a similar way to terrestrial macromolecules. Concentrated sulfuric acid is a chaotropic solvent that is known to disrupt hydrogen bonds that are crucial for the maintenance of the 3D structure of terrestrial macromolecules (e.g.^54^). Whether the denaturing effect of concentrated sulfuric acid is an insurmountable obstacle in the formation of functional macromolecules of life in general, no matter its chemical basis, is a topic of future work.

## Materials and methods

The set of 17 L-homodipeptides was custom synthesized at $\ge$ 95% purity by CPC Scientific Peptide Company Inc. (https://cpcscientific.com/). Note that the QQ dipeptide was difficult to synthesize due to the formation of cyclic structure and its instability during lyophilization. The QQ dipeptide has been obtained at 88% purity. We purchased three L-homodipeptides (AA: cat.no. A9502, GG: cat. no. G1002 and EE: cat. no. G3640) from Millipore-Sigma with $\ge$ 98% purity. All dipeptides have been used without further purification. We used D_2_SO_4_ from ACROS Organics (sulfuric acid-d_2_ for NMR, 98 wt% in D_2_O, 99.5 + atom % D) and D_2_O (deuteration degree min 99.9%) from MagniSolv.

We prepared our NMR samples by dissolving each dipeptide into 500–700 uL of solvent D_2_SO_4_ in D_2_O in glass vials. Most of the dipeptide solutions have been heated to 80 ºC for a few minutes to promote dissolution. We used 30 mg of compounds for the 1D ^1^H and ^13^C NMR. We transferred the solution to 5 mm NMR tubes and stored the tubes for 12–18 h before NMR measurements. After NMR measurements we stored the solutions in the NMR tubes, where the storage room temperature varied from about 18–24 ºC.

To acquire NMR data, we used a Bruker Avance III-HD 400 MHz spectrometer equipped with a Prodigy liquid nitrogen cryoprobe (BBO) at 25 °C. We acquired 1D ^1^H and ^13^C NMR spectra to confirm the structures and hence stability of the compounds in 98% w/w and 81% w/w D_2_SO_4_ in D_2_O. In all cases we locked on D_2_SO_4_. The D_2_SO_4_ peak is at 11.48 ± 0.02 ppm in 98% w/w D_2_SO_4_ and at 11.99 ± 0.02 ppm in 81% w/w D_2_SO_4_. As a control and to further confirm the stability and the overall structural integrity of each of the dipeptides we have acquired 1D ^1^H and ^13^C NMR spectra in pure D_2_O as well. See Supplementary Information for the list of all associated datasets.

We used MNova software (Mestrelab Research) to process and analyze the NMR data^55^. The original data for all NMR experiments are available for download as Supplementary Datasets from Zenodo at https://zenodo.org/records/11223995.

### Supplementary Information

Supplementary Information.

## Data Availability

Original data are deposited in Zenodo data repository at https://zenodo.org/records/11223995. The authors are also willing to provide the original datasets on request.
