# Supplementary material for: General instability of dipeptides in concentrated sulfuric acid as relevant for the Venus cloud habitability
Source: Sci Rep. 2024 Jul 24;14:17083. doi: 10.1038/s41598-024-67342-w (PMC11269616; doi:10.1038/s41598-024-67342-w)
Supplement: Supplementary file 1 — Supplementary Information. [file 41598_2024_67342_MOESM1_ESM.docx]

**Supplementary Information**

**General Instability of Dipeptides in Concentrated Sulfuric Acid and Implications for the Habitability of Venus’ Clouds**

Janusz J. Petkowski ^1,2,3#,*^, Maxwell D. Seager^4,5,#^, William Bains ^1,6,7^, Sara Seager ^1,5,8,9#^

^1^ Department of Earth, Atmospheric and Planetary Sciences, Massachusetts Institute of Technology, 77 Massachusetts. Avenue., Cambridge, MA 02139, USA

^2^ Faculty of Environmental Engineering, Wroclaw University of Science and Technology, 50-370 Wroclaw, Poland

^3^ JJ Scientific, Mazowieckie, Warsaw 02-792, Poland.

^4^ Department of Chemistry and Biochemistry, Worcester Polytechnic Institute, Worcester, MA 01609, USA

^5^ Nanoplanet Consulting, Concord, MA 01742, USA

^6^ School of Physics & Astronomy, Cardiff University, 4 The Parade, Cardiff CF24 3AA, UK

^7^ Rufus Scientific, Melbourn, Herts SG8 6ED, UK

^8^ Department of Physics, Massachusetts Institute of Technology, 77 Massachusetts. Avenue., Cambridge, MA 02139, USA

^9^ Department of Aeronautics and Astronautics, Massachusetts Institute of Technology, 77 Massachusetts. Avenue., Cambridge, MA 02139, USA

******* Correspondence: [jjpetkow@mit.edu](mailto:jjpetkow@mit.edu)

# contributed equally to this work

­**Supplementary Figures:**

**
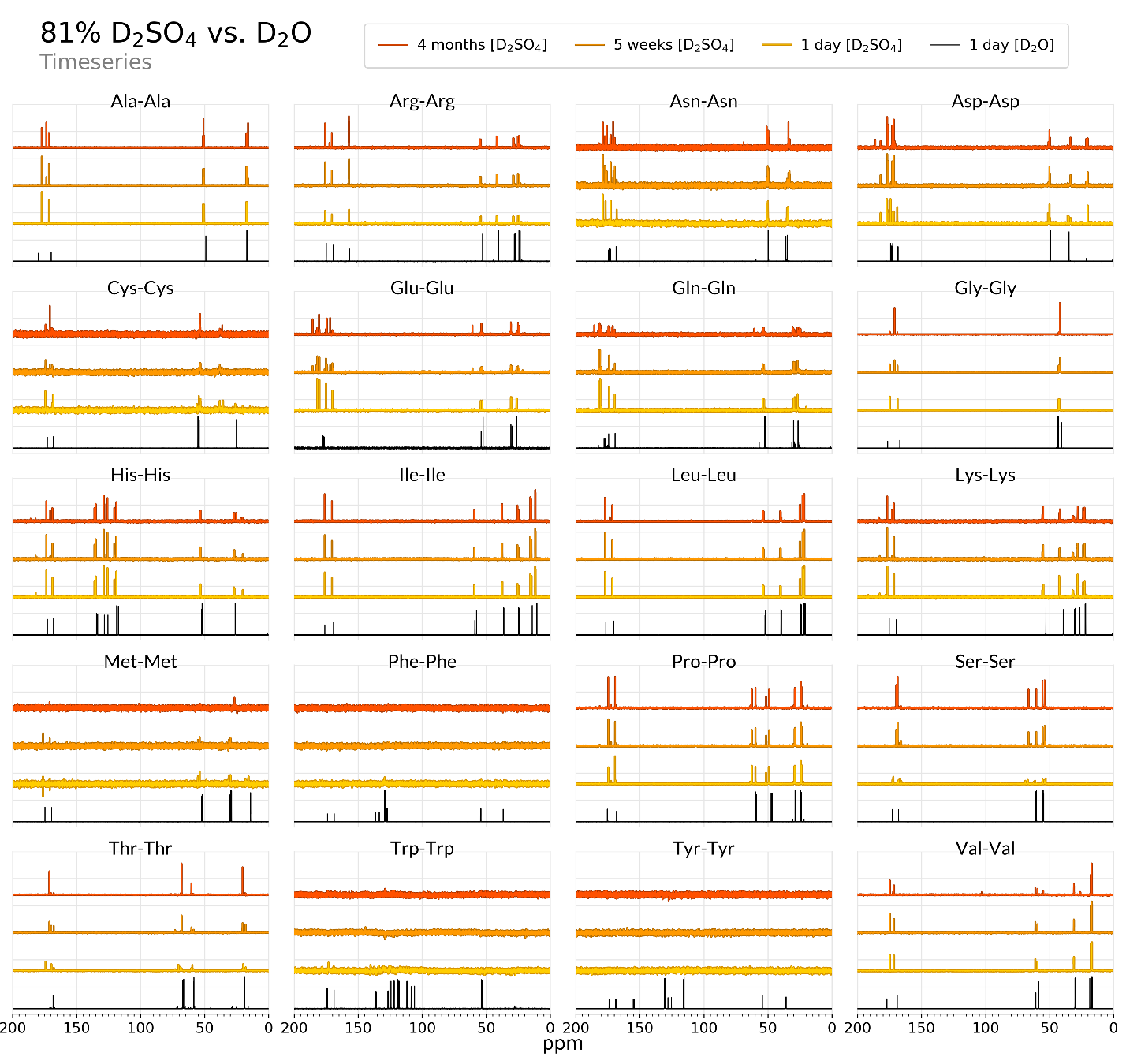
Figure S1.** Reactivity of the 20 homodipeptides in 81% w/w concentrated sulfuric acid at room temperature. Time series of ^13^C NMR spectra of the homodipeptides with colors indicating the time incubated in concentrated sulfuric acid at room temperature: yellow = 1 day, orange = 5 weeks, red = 4 months, compared to the spectra collected after 1 day incubation in pure D_2_O (black). Most of the dipeptides are unstable in 81% w/w sulfuric acid, with few exceptions. Dipeptides II, LL, VV, PP, RR and KK resist hydrolysis in 81% w/w sulfuric acid for at least 5 weeks.

**
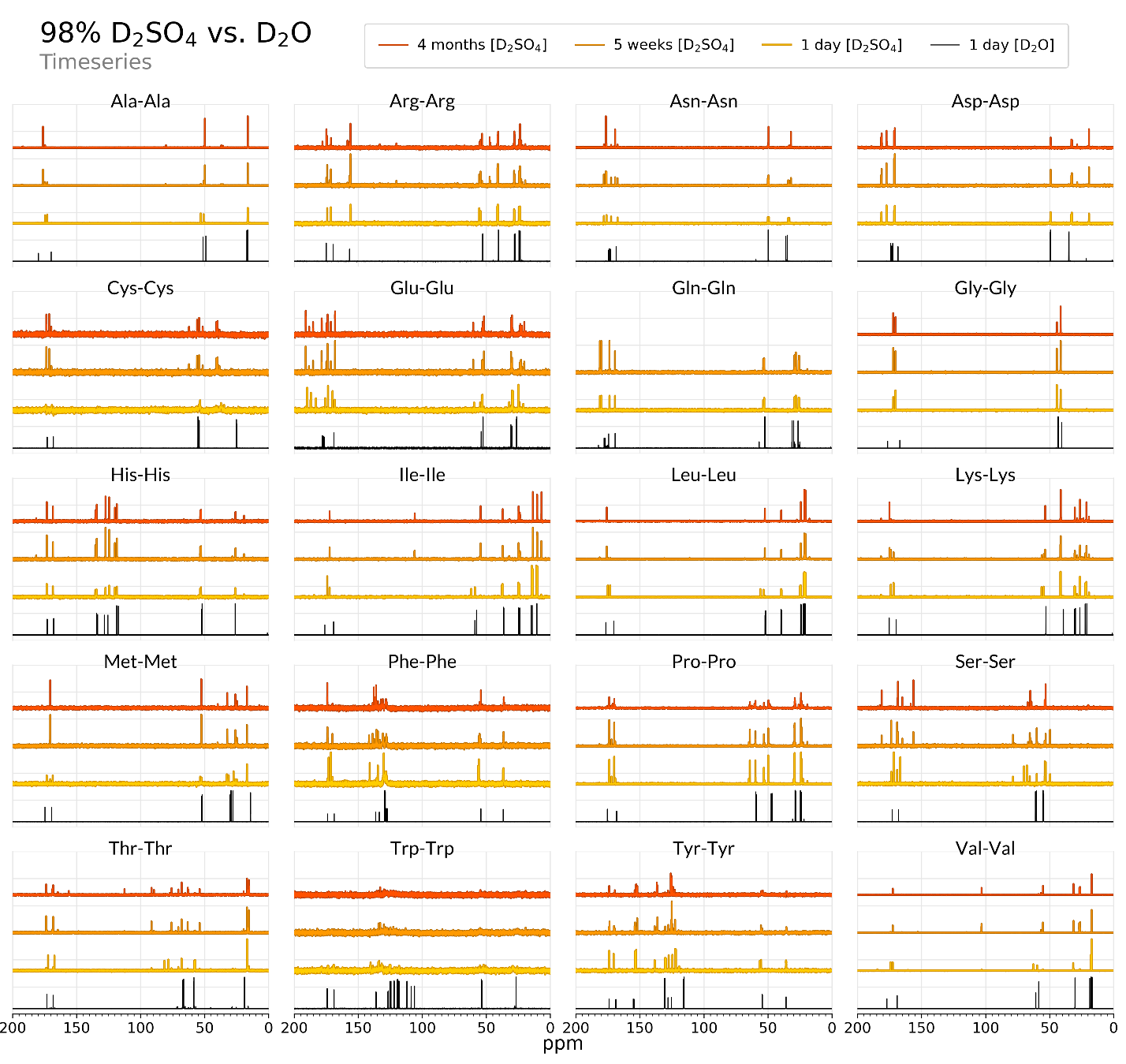
Figure S2.** Reactivity of the 20 homodipeptides in 98% w/w concentrated sulfuric acid at room temperature. Time series of ^13^C NMR spectra of the homodipeptides with colors indicating the time incubated in concentrated sulfuric acid at room temperature: yellow = 1 day, orange = 5 weeks, red = 4 months, compared to the spectra collected after 1 day incubation in pure D_2_O (black). Most of the dipeptides are unstable in 98% w/w sulfuric acid, with few exceptions. Dipeptides HH and GG resist hydrolysis in 98% w/w sulfuric acid for at least 4 months.

**
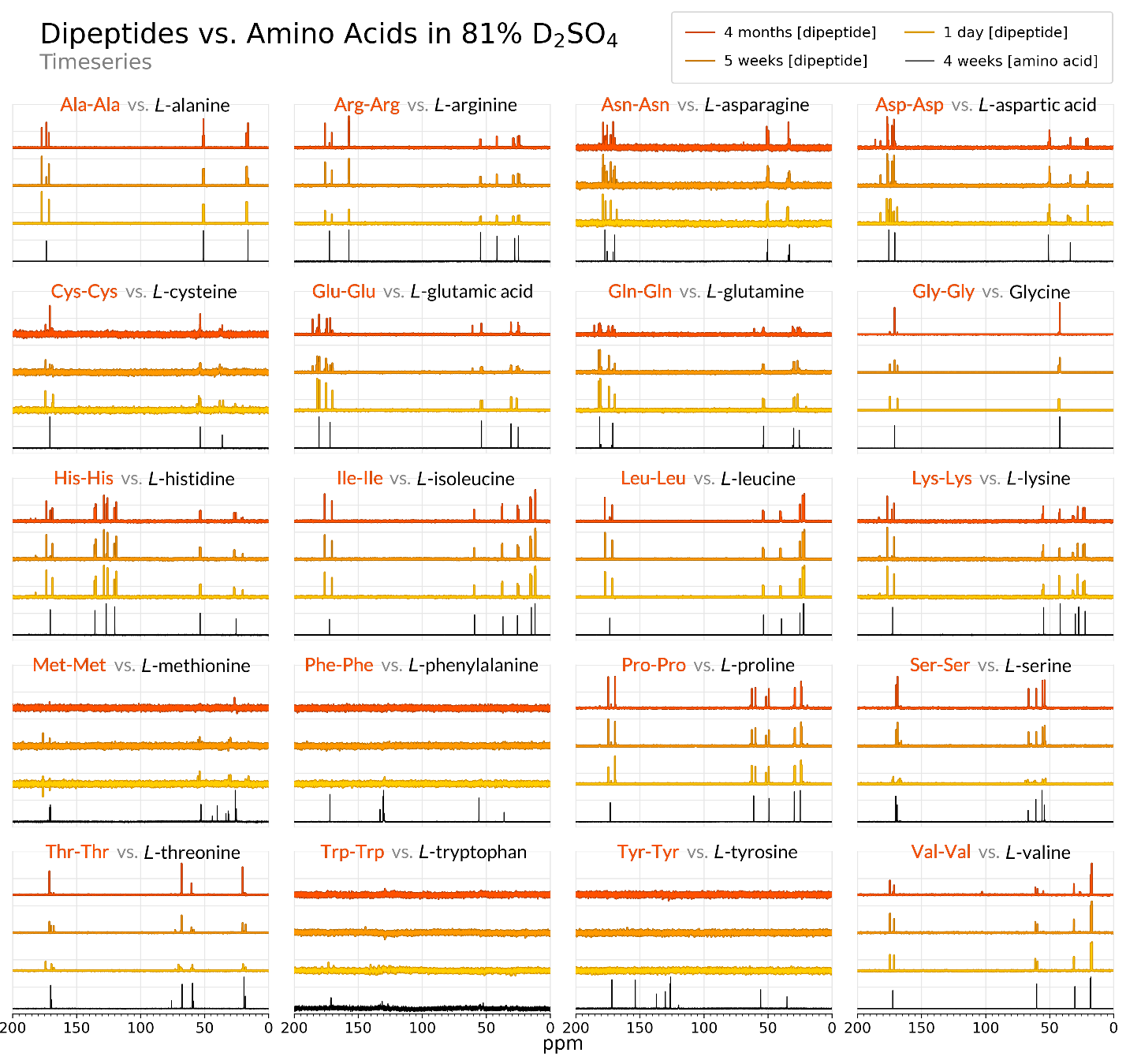
Figure S3.** Reactivity of the 20 homodipeptides in 81% w/w concentrated sulfuric acid at room temperature. Time series of ^13^C NMR spectra of the homodipeptides with colors indicating the time incubated in concentrated sulfuric acid at room temperature: yellow = 1 day, orange = 5 weeks, red = 4 months, the 1-month-incubated single amino acid (black) ^1^. Most of the dipeptides are unstable in 81% w/w sulfuric acid and undergo a well-known process of acid-catalyzed hydrolysis and the release of the monomeric amino acid residues.

**
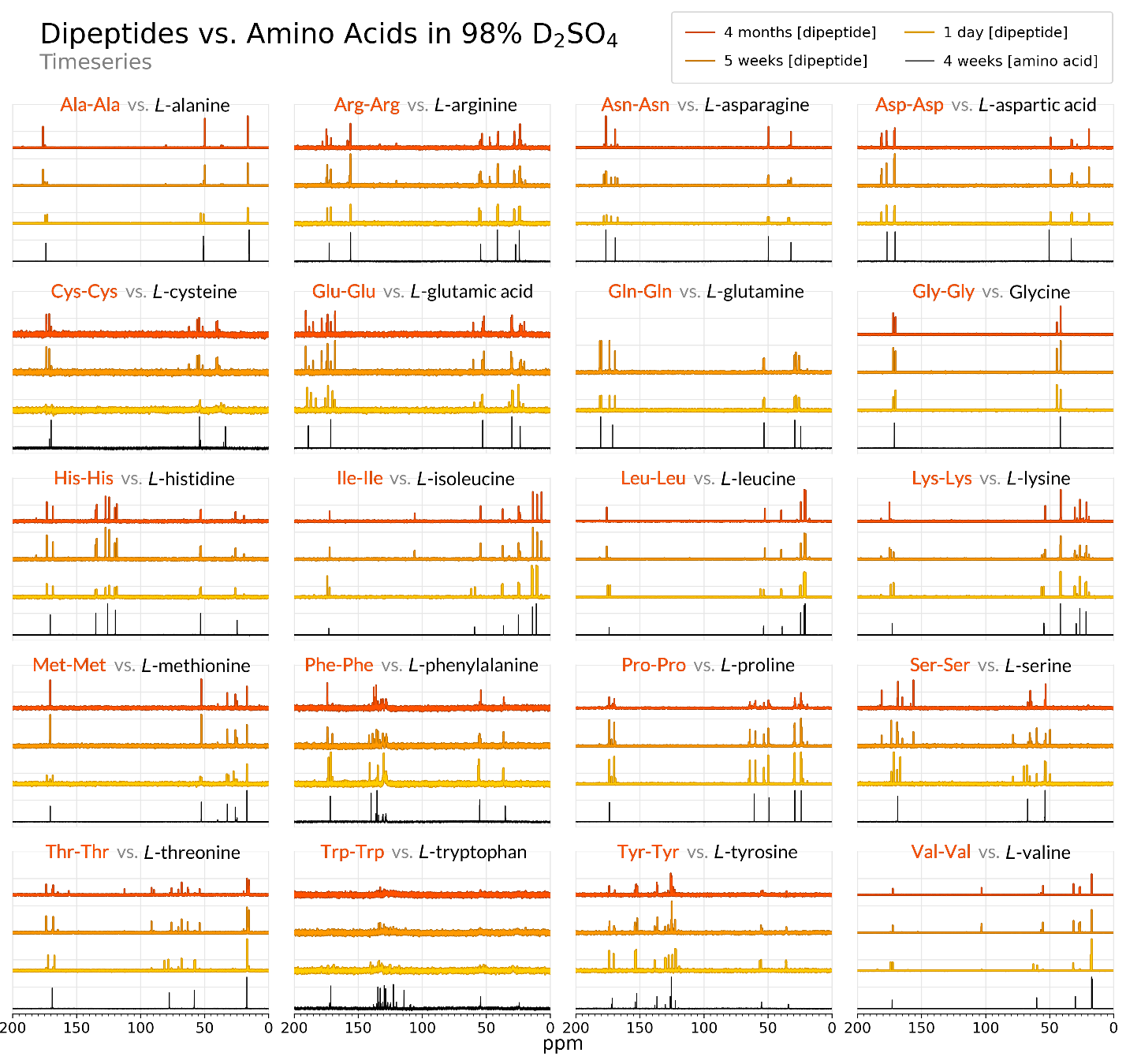
Figure S4.** Reactivity of the 20 homodipeptides in 98% w/w concentrated sulfuric acid at room temperature. Time series of ^13^C NMR spectra of the homodipeptides with colors indicating the time incubated in concentrated sulfuric acid at room temperature: yellow = 1 day, orange = 5 weeks, red = 4 months, the 1-month-incubated single amino acid (black) ^1^. Most of the dipeptides are unstable in 98% w/w sulfuric acid and undergo solvolysis leading to different products than the canonical acid-catalyzed hydrolysis.

**Supplementary Datasets:**

*Supplementary Dataset S1:* The original 1D ^1^H and ^13^C NMR data collected in 81% w/w and 98% w/w concentrated sulfuric acid, at all tested time intervals, for all 20 homodipeptides. All data can be downloaded from Zenodo at: <https://zenodo.org/records/11223995>.

*Supplementary Dataset S2:* The original 1D ^1^H and ^13^C NMR data collected in D_2_O, for all 20 homodipeptides. All data can be downloaded from Zenodo at: [https://zenodo.org/records/11223995](https://zenodo.org/uploads/11223995).

**Supplementary References:**

1. Seager, M. D., Seager, S., Bains, W. & Petkowski, J. J. Stability of 20 Biogenic Amino Acids in Concentrated Sulfuric Acid: Implications for the Habitability of Venus’ Clouds. *Astrobiology* **24**, 386–396 (2024).
